# Comparative study of renal drainage with different ureteral stents subject to extrinsic ureteral obstruction using an in vitro ureter-stent model

**DOI:** 10.1186/s12894-021-00865-w

**Published:** 2021-07-14

**Authors:** Yaniv Shilo, Jonathan Modai, Dan Leibovici, Ishai Dror, Brian Berkowitz

**Affiliations:** 1 Department of Urology, Kaplan Medical Center, 7661041 Rehovot, Israel; 2 Department of Earth and Planetary Sciences, Weizmann Institute of Science, 7610001 Rehovot, Israel

**Keywords:** Tandem stents, Metal stents, Endopyelotomy, Stent failure, Colloids

## Abstract

**Background:**

To compare the efficacy of different ureteral stents subject to extrinsic ureteral obstruction (EUO), in a controlled in vitro stented ureter experiment.

**Methods:**

We employ an in vitro ureter-stent experimental set-up, with latex tubing simulating flexible ureters attached to vessels simulating renal units and bladders. The flow behavior of five ureteral stents—polymeric 8F, tandem 6F, tandem 7F, endopyelotomy and metal—was tested under a ureteral deformation configuration of 40°, with 2000 g external force over a 3.5 cm length of the ureter. A constant fluid flow was applied through the ureter-stent configurations, and pressure fluctuations in the renal unit were monitored. We considered a renal unit pressure of 10 cmH_2_O or flow discontinuation in the bladder as stent failure. Urine containing debris was mimicked by use of a colloidal solution.

**Results:**

Of all assessed ureteral stents, under EUO conditions, only the single 8F stents remained patent throughout the length of the experiment. All other stents—tandem 6F and 7F, single 7F, metal and endopyelotomy—displayed limitations.

**Conclusions:**

Tandem and metal stents show no superiority over large luminal polymeric stents for EUO treatment in this in vitro model. Larger luminal stents offer excellent resistance to external pressure and allow adequate colloidal flow. The need for frequent exchange and bladder irritation should also be considered in the choice of stent configuration for treatment of kidney drainage under EUO.

**Supplementary Information:**

The online version contains supplementary material available at 10.1186/s12894-021-00865-w.

## Background

Extrinsic ureteral obstruction (EUO) is a common situation that may be related to different etiologies. EUO is often due to pelvic malignancy—either primary tumor or metastatic disease—while other causes are due to benign etiologies that include ovarian cysts, retroperitoneal fibrosis, benign pelvic masses, surgical complications, and possibly radiation damage [1–3]. As such, patients suffering from EUO usually have poor prognosis and short life expectancy. The main complication of EUO is renal failure due to ureteral obstruction, leading to hydronephrosis; drainage of the relevant kidney with either ureteral stent or nephrostomy tube is imperative.

Double-J ureteral stents generally represent a more appealing drainage option, as nephrostomy tubes carry the inconvenience of an external urine bag. However, as many as ~ 50% of ureteral stents placed to treat EUO fail (in terms of drainage, leading to renal failure and hydronephrosis), often within weeks [1]. While the exact mechanism(s) for stent failure is unclear, large caliber [2, 3], tandem [4–9] and metal [10–17] stent designs have been suggested to overcome this problem. Several studies—mostly retrospective—have compared the success rates of different stents under EUO, but to date, no clear conclusion defining the stent(s) that offer(s) the best outcome can be drawn.

Here, we examined potential differences in behavior and the likelihood of stent failure among large diameter (8F) and tandem (6F, 7F) polymeric stents, a metal stent, and a polymeric endopyelotomy stent (7F/14F). We employed an in vitro experimental setup [18] to investigate the influence of both ureteral deformation and compression, and the synergistic effect of colloid presence in the fluid, on stent patency.

## Materials and methods

### In vitro stent-ureter-kidney model

**Fig. 1:**
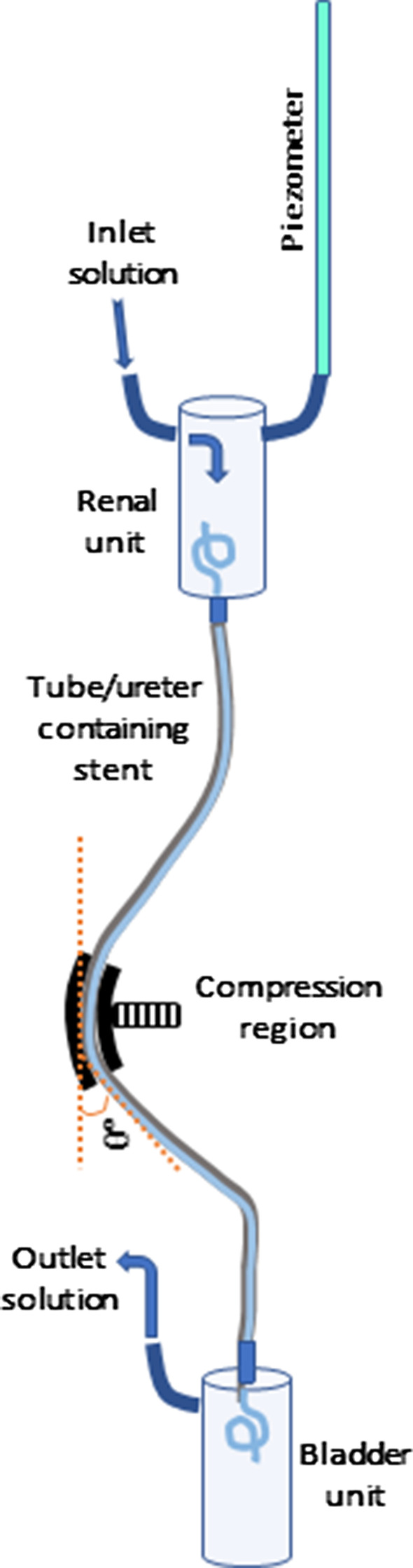
Schematic showing the experimental setup, with details of a single ureter-stent unit, including of the angle of deformation, *θ*°

We employed an in vitro ureter-stent experimental set-up described in detail elsewhere [18]. Briefly, we used natural latex tubing (inner diameter 4.76 mm, wall thickness 0.79 mm) to simulate flexible ureters attached to glass vessels representing renal units and bladders, and examined flow behavior in ureteral stents. The experimental set-up (single-unit) is shown schematically in Fig. 1; the laboratory system enabled simultaneous running of 12 stent units, housed within a temperature-controlled chamber (37 °C). The ureter-stent configuration simulated external ureteral pressure causing ureteral deformation to *θ* = 40° (Fig. 1). EUO was simulated by semi-circular compression of length ~ 3.5 cm located in the region of maximum deformation; the ureter/stent configuration provided an applied force of 2000 g (≈ 19.6 N). In the context of the colloidal fluid experiments considered here, and given our findings ([18] and below) that stent failure is due to colloid accumulation particularly in the region of compression, the EUO location along the ureter (more proximally or distally) is not significant. This was confirmed by a preliminary experiment (not shown), and supported by computational fluid dynamics simulations [19]. Further discussion justifying the choice of materials and applied force appears in the Additional file 1.

**Fig. 2:**
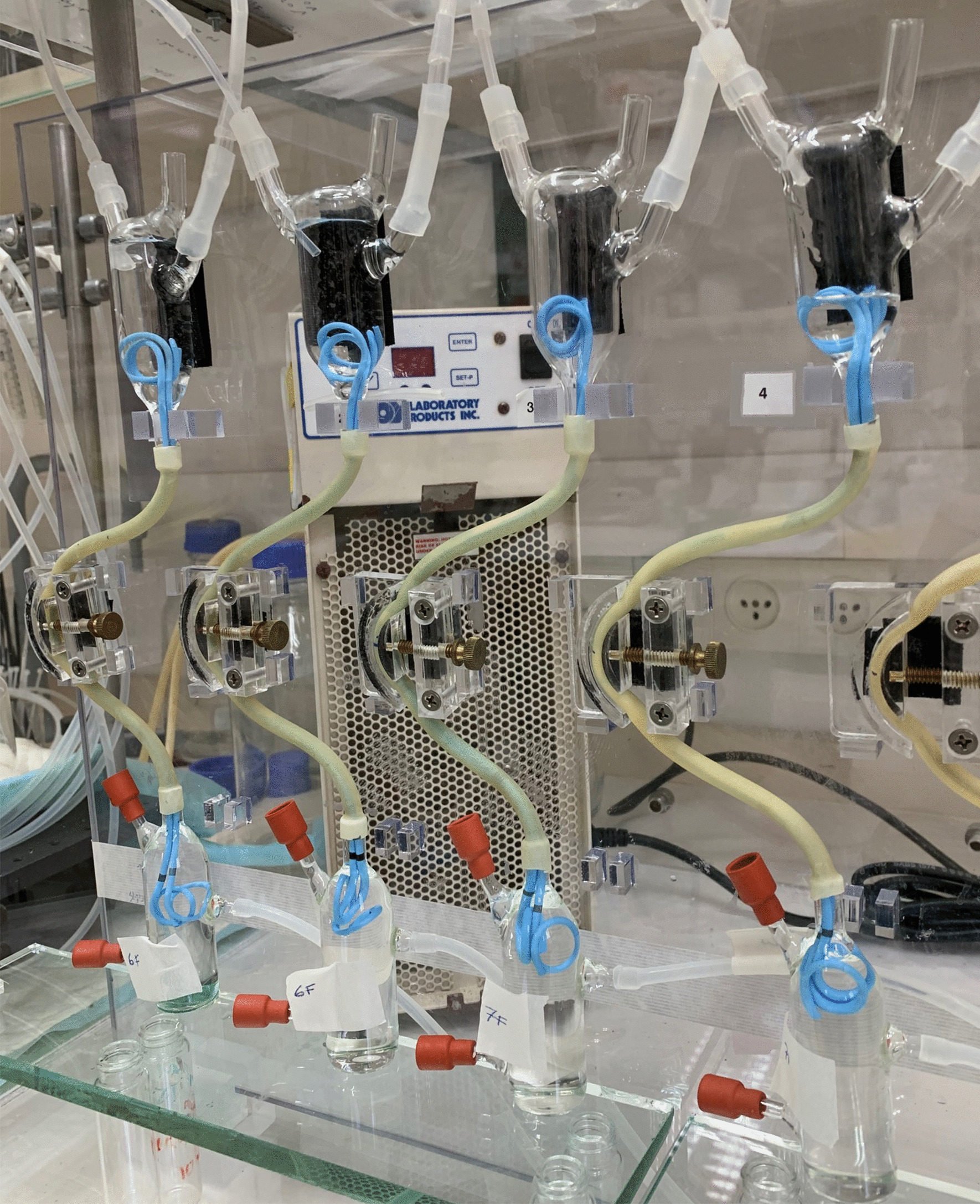
Photograph of part of the 12-unit experimental systems, showing two tandem 6F and two tandem 7F stent-ureter units, under deformation and compression

Each ureter-stent unit contained double-J stents—either a single 8F stent, tandem (pairs of) 6F or 7F stents (Boston Scientific® Percuflex Plus), a single Resonance® metallic stent (6F, Cook Medical), or a single 7F/14F endopyelotomy stent (Boston Scientific, Retromax Plus). An example of four such ureter-stent units is shown in Fig. 2.

In each ureter-stent unit, the proximal and distal pigtails were inserted into renal unit and bladder vessels, respectively. For the endopyelotomy stent, the 14F section was placed at the distal side, with compression acting over part of this region. A constant flow rate was prescribed in each of the 12 units, via a 12-channel peristaltic pump (Ismatec® Model-IPC12-Channel pump); the fluid volume in the renal unit vessel damped small pressure fluctuations induced by the pump. In all experiments, the volumetric flow rate in each renal unit was set at 30 mL/h, with maximum deviation at the system outlet of 1%; this flow rate is within the general range of flow rates, 25–50 mL/h [20]. Fluid pressure in each renal unit was monitored with a piezometer.

### Flow experiments and colloid solution

Fluid flow (saline solution; 9 g NaCl/1 L double distilled water) in each of the five stent configurations (8F, tandem 6F, tandem 7F, Resonance®, endopyelotomy) was first tested over a range of compressive forces up to 2000 g, following tests for single stents [18] (see Additional file 1), to confirm that deformation and pressure do not lead to stent failure. Subsequently, as in Shilo et al. [18], we employed a colloidal solution containing chicken albumin from egg white powder (see Additional file 1).

Here, two sets of (12 ureter-stent unit) experiments (348 h duration; determined by the experimental results presented below) were completed with stented ureters under deformation and compression, together with the colloidal solution. In total, experiments comprised (i) four replicates each of the tandem 6F, tandem 7F and Resonance® stents, (ii) two replicates each of the endopyelotomy (tested for “completeness”, though of less clinical interest) and 8F stents (in addition to 4 similar replicates with the 8F stent reported in Shilo et al. [18]), and (iii) two replicates each of the control ureter-stent units, with tandem 6F, tandem 7F, Resonance® and 8F stents, with no deformation or compression. Throughout the experiments, fluid heights in the piezometers varied at ± 1 mm (± 0.1 cmH_2_O) due to the action of the peristaltic pump. Fluid heights in each piezometer were monitored over time for stent failure, which was defined as renal unit pressure reaching 10 cm/H_2_O (and ultimately complete obstruction of fluid flow).

## Results

We showed previously [18, 21] that deformation/compression alone, using a solution without colloids, are not generally sufficient to lead to stent failure under EUO, in tests with 4.8F, 6F, 7F and 8F stents. In particular, we confirmed [18] that the 8F stent shows no decrease in flow (or increase in renal pressure) up to 5000 g compression. In the current experiments, we found, too, in preliminary measurements, that like the single 8F stent, the tandem 6F and 7F stents, the Resonance® metallic stent, and the 7F/14F endopyelotomy stent are similarly unaffected by realistic deformation/compression.

Results of the experiments with the colloidal suspension are summarized in Table 1, with all units, except the controls, kept under deformation/compression. Notably, all control units for the (unperturbed) stented ureters—tandem 6F, tandem 7F, Resonance®, 8F stents—remained open.

**Table 1:** Time to stent failure (blockage), in hours, under deformation of 40° and compression of 2000 g in the region of the external ureteral obstruction

Replicate	Stent				
	Tandem 6F (time to blockage, hour)	Tandem 7F (time to blockage, hour)	Resonance^®^ (time to blockage, hour)	Endopyelotomy 14F (time to blockage, hour)	8F^†^ (time to blockage, hour)
1	132	133.5	243.5	300.5	–^‡^
2	274	103	124.5	235.5	–^‡^
3	325	329	224		
4	–^‡^	303.5	–^‡^		

Entries with “–” indicate no failure. All failure times are rounded to the nearest half hour

^†^The same result (no blockage) for 4 similar replicates with the 8F stent was reported in [18]

^‡^Experiment duration of 348 h

The 8F stented ureter was the only system to remain open in all cases. Results for the four replicates of the 8F stent system tested previously [18], using a slightly smaller diameter ureter tube, are relevant here because *in the vicinity of the EUO*, the stent lumen, not the ureter lumen, essentially controls flow; urine flowing within the ureter is likely diverted through the stent, via side holes, to bypass the region of compression (see Additional file 1). Thus, total ureter-stent blockage and pressure buildup are controlled, *ultimately*, by the stent lumen.

From Table 1, the endopyelotomy and tandem 7F stents failed in all replicates, with average times of 268 h (235.5, 300.5 h) and 217 h (103, 133.5, 303.5, 329 h), respectively. The tandem 6F stents failed in three replicates at an average time of 244 h (132, 274, 325 h); one replicate continued to drain freely. Similarly, the Resonance® stents failed in three replicates at an average time of 197 h (124.5, 224, 243.5 h) and one replicate continued to drain. Throughout the experiments, small rises (up to 1–1.5 cm) —and then falls—were observed in many piezometers, including in the control units. The Resonance® and tandem 6F and 7F stents exhibited even broader pressure rises and falls, with some units rising once or twice up to 6 cm before then falling. This behavior was evidently a result of colloid accumulation leading to partial clogging and pressure build-up, and then break-up and release and/or rearrangement of (some of) the accumulated colloids in the stented ureter system (see Discussion below). Once pressure rose above ~ 6 cm, it never decreased and generally continued to rise quickly to > 10 cm.

## Discussion

EUO prevalence is growing steadily and continues to be a major treatment challenge for the urologist [3]. Different stent types and configurations have been reported in an effort to adequately drain kidneys and prevent complications. Over recent years, tandem ureteral stents [4–9], metal stents [10–15] or metal-mesh stents [16, 17] have been advocated as the preferred drainage method under EUO. However, few “head-to-head” studies comparing all of these options are available, which can be attributed to limitations that include relatively low numbers of patients with EUO that can be recruited at a single center, different etiologies responsible for EUO, and differences in ideal stent preferences between medical centers. We present here the first comparison of a wide variety of different ureteral stents subjected to EUO in an in vitro model of a stented ureter. Our experiments indicate that neither tandem ureteral stents nor metal stents offer any clear advantage over large luminal polymeric stents in preventing stent failure, although they appear more effective than single, small luminal polymeric stents (comparing failure times to those reported in [18]). In addition, endopyelotomy stents show no superiority over large luminal polymeric stents under EUO conditions of deformation, external ureteral compression and fluids containing colloids.

In a retrospective study, Askawa et al. [13] compared polymeric stents to Resonance® stents in patients with EUO. A total of 54 ureters in 35 patients were drained with polymeric stents, and 72 ureters in 57 patients were treated with Resonance® stents. Overall stent patency for both groups was 70%, while Resonance® stents showed higher patency rates when compared to polymeric stents after one year of follow-up (78% vs. 61%). This difference did not reach statistical significance in multivariate analysis. The authors [13] concluded that the Resonance® stent is superior to polymeric stents as it better resists external compression forces, so that it should be the first choice to drain EUO. Another comparative study by Liu et al. [7] evaluated prospectively, in a non-randomized fashion, 104 patients with EUO using single or tandem 7F polymeric stents based on patient preference. Stent patency duration among 63 renal units drained with tandem stents was 214 days, in comparison to 176 days among 94 patients with single stents. The authors concluded that tandem polymeric stents are superior to single stents.

These authors [7, 13] suggested that a key etiology responsible for stent failure under EUO is external compression forces exerted over the ureter, resulting in deformation, kinking and closure of the stent and ureter lumina. While it may be intuitive to expect that polymeric stents show poor resistance to external ureteral pressure, we showed previously [18, 21] that this is not the case via in vitro experiments. Polymeric stents (4.8F to 8F) were subjected to both ureteral deformation and increasing external forces (compression) of up to 5000 g; the 4.8F stent failed at high compression, while the 6F, 7F and 8F stents remained patent throughout. We concluded [18, 21] that only unrealistic external pressure forces may lead to stent obstruction and therefore external pressure/deformation as sole etiology for stent failure is less reasonable. Two biases should be noted in the two above-mentioned studies [7, 13] comparing polymeric stents to either tandem or metal stents. First, the luminal size of the polymeric stents that were compared to the Resonance® stent was not mentioned; use of a relatively small luminal size might have influenced the results. Second, the tandem and single stents in the comparative study [7] were all 7F. Clearly, the likelihood for a stent to remain patent is higher when comparing two stents of the same size in one ureter to a different ureter drained with only one stent of the same size. A valuable comparison would have been between 7F tandem stents and a single 8F polymeric stent.

In the current experiment, only the 8F stent remained patent consistently throughout the experiment duration, while all other stent configurations—tandem 6F and 7F, Resonance®, endopyelotomy—generally failed. The exact mechanism in which ureteral stents fail under EUO is unclear, although several possible risk factors have been suggested [2, 7], including stent incrustation/encrustation due to mineralization, blockage by debris accumulation, loss of ureteral peristalsis, tissue growth through stent side holes and/or stent buckling. As described previously [21], colloidal fluid can play a critical synergetic role with deformation and compression in the occurrence of stent failure. Assessment of different luminal polymeric sizes stents subjected to EUO in an in vitro model resulted in stent failure of the smaller luminal sizes (4.8F and 6F) as opposed to the larger sizes (7F and 8F) that remained open. We note, too, that tandem stents have been reported to be effective in some clinical settings under EUO [5, 7, 9, 22]. We speculate that this may be due to movement between the stents, which may reduce colloid aggregation and accumulation of colloids, as well as to the possibility that tandem stents maintain a space between the stents to allow urine flow even subject to ureter and stent lumina obstruction.

The time to failure variation of each stent type and configuration is likely related to the “random” nature of colloid accumulation over time. In many cases, we noted steady rising renal pressure and then sudden pressure drops, which were most likely due to accumulation and subsequent release of colloids. Whether mobilization of patients with EUO assists in prevention of colloid accumulation has yet to be proved. We emphasize that we make no claim regarding actual times to stent failure in clinical settings, nor do we consider EUO progression over time. Rather, the experiment was designed to work with a specific colloidal concentration and examine the relative times to failure (or not) among the different stent sizes and configurations.

Existing literature and the results reported here indicate that larger-lumen stents are less likely to become occluded with debris, due to the higher flow rates within them. Because urine flow in a ureter-stent system is generally laminar [18], *illustrative* flow calculations can be derived from Poiseuille’s law. We note, too, that while the relative contributions of stent and ureter lumina to overall flow remain poorly understood, the stent lumen likely controls flow behavior particularly in the vicinity of the EUO where the ureter lumen is obstructed, which ultimately affects overall flow through the ureter-stent system; we therefore focus on stent flow in this region. The volumetric flow, *Q*, through a tube is *Q* = (π*Pr*^4^)/(8*ηl*), where the dynamic viscosity for urine is [23] *η* ≈ 8.5 × 10^−4^ Pa·s at 37 °C and *r* is the internal radius. We assume a straight stent length *l* = 24 cm (no pigtails), and total stent radii of *r* = 0.8, 1.0, 1.17 and 1.33 mm (corresponding to 4.8F, 6F, 7F and 8F stents, respectively). We modify *r* to account for wall thickness (measured [21] as 0.22 mm for the 4.8F, 0.4 mm for 6F, 7F, 8F stents), and impose a renal unit pressure of 1 cmH_2_O (*P* = 98.0665 Pa). Calculations of *Q* in different stent configurations are given in Table 2. Notably, increasing stent luminal diameter, e.g., from 6 to 8F, increases volumetric stent flow by a factor of 5.9. In this context, consideration of tandem stents does not necessarily lead to increased volumetric flow rates. For example, tandem 6F stents have an effective, combined cross-sectional area of 6.28 mm^2^, while a single 8F stent has an effective cross-sectional area of 5.55 mm^2^; and yet, the volumetric flow in a single 8F stent is still a factor of 2.9 higher than in tandem 6F stents (and a factor of 1.1 higher than tandem 7F stents). These results are controlled by the strong effects of frictional forces on the stent walls, which vary significantly as a function of radius—critically, *Q* varies with the factor *r*^4^.

**Table 2:** Poiseuille flow calculations for flow in stents

–	Stent						
	Single 4.8F	Tandem 4.8F	Single 6F	Tandem 6F	Single 7F	Tandem 7F	Single 8F
Flow rate (mL/h)	77	154	88	176	235	470	516
Internal stent radius (mm)	0.58	–	0.60	–	0.77	–	0.93
Cross-sectional area (mm^2^)	2.01	4.02	3.14	6.28	4.30	8.60	5.55

The limitations of our study are related to the model design [18, 21]; see Additional file 1 for discussion regarding latex tubing, EUO shape and pressure, and type of colloidal solution (rather than “artificial urine”). Clearly, an in vitro experimental study cannot account for all physiological properties in the human in vivo environment. As such, the findings reported here do not correlate *directly* to clinical findings, particularly in terms of times to stent failure. However, our systematic analysis among ureter-stent configurations yields important relative comparisons of their failure dynamics. The latex tubing simulating a ureter has physical characteristics different than a real ureteral wall, and the lack of (even minimal) peristaltic motion may have some effect on the results (although we note that loss of ureteral peristalsis occurs frequently with stenting [2, 7, 24]). Moreover, dynamic ureteral responses caused by indwelling stents and EUO will likely further affect the flow dynamics over time, such as reflux or retrograde pressure transmission through and/or around the stent. The results here also are a function of the type and concentration of colloid material, and the use of solution containing no other salts, enzymes and organic material. As such, stent mineralization, various colloid aggregation properties, and biological activity remain unaccounted for. Moreover, the occurrence of blockage and the actual times to blockage (and variability among replicate experiments) are also affected by colloid concentration. Notwithstanding the above, this is the first study to systematically measure effects of colloidal fluid flow in a comparative study of single, tandem, metal, and endopyelotomy stents in stented-ureter systems, both under and without EUO.

## Conclusions

With the increased prevalence of EUO and multiple choices of ureteral stent configurations for drainage, comparison of treatment options is important. While tandem and metal stents have been suggested to be superior to single polymeric stents, our results show that large luminal stents offer no inferior patency rates and even better ones, at least in this in vitro model. Larger luminal stents offer excellent resistance to external ureteral pressure and allow adequate urine flow with colloids. In all cases, though, it should be emphasized that "ideal" stent drainage for EUO should take into consideration not only stent patency, but also patient quality of life, frequency of stent exchange, and associated costs that are influenced by stent size and composition.

## Supplementary Information

**Additional file 1.** Ureter, obstruction and colloidal solution set-up for the in vitro experiments.

## Data Availability

All data generated or analysed during this study are included in this published article and its supplementary information file.

## Abbreviations

EUO: Extrinsic ureteral obstruction
*η*: Dynamic fluid viscosity
*l*: Tube length
*P*: Pressure difference between tube inlet and outlet
*Q*: Volumetric fluid flow rate
*r*: Ureter radius
