# Supplementary material for: Comparative study of renal drainage with different ureteral stents subject to extrinsic ureteral obstruction using an in vitro ureter-stent model
Source: BMC Urol. 2021 Jul 14;21:100. doi: 10.1186/s12894-021-00865-w (PMC8281631; doi:10.1186/s12894-021-00865-w)
Supplement: Supplementary file 1 — Additional file 1. Ureter, obstruction and colloidal solution set-up for the in vitro experiments. [file 12894_2021_865_MOESM1_ESM.docx]

**SUPPLEMENTARY INFORMATION**

**Comparative study of renal drainage with different ureteral stents subject to extrinsic ureteral obstruction using an *in vitro* ureter-stent model**

**Yaniv Shilo^1^, Jonathan Modai^1^, Dan Leibovici^1^, Ishai Dror^2^ and Brian Berkowitz^2^**

1. Department of Urology, **Kaplan Medical Center, Rehovot** 7661041 **Israel (**[drshiloy@gmail.com](mailto:drshiloy@gmail.com); **jmodai@gmail.com;** DanLe1@clalit.org.il**)**

**2. Department of Earth and Planetary Sciences, Weizmann Institute of Science, Rehovot 7610001, Israel (**[ishai.dror@weizmann.ac.il](mailto:ishai.dror@weizmann.ac.il)**;** [brian.berkowitz@weizmann.ac.il](mailto:brian.berkowitz@weizmann.ac.il)**)**

As noted in the main text, any *in vitro* experimental study has limitations, and cannot account for all physiological properties in the human *in vivo* environment. We expand here on background information and results of previous experimental analyses that justify the choices of latex tubing, EUO shape and pressure, and type of colloidal solution employed in our current study. The *in vitro* ureter-stent experimental set-up is described in further detail by Shilo et al. [1, 2].

**MATERIALS AND METHODS**

*In Vitro Ureter Model*

The flexible ureter was simulated by natural latex tubing (Latex-Tubing.com; inner diameter 4.76 mm (3/16 inch), wall thickness 0.79 mm (1/32 inch)). **This tubing was determined to feel most similar to human ureters upon testing by different senior urologists, who examined several types of tubing material and wall thicknesses. The use of latex tubing excludes effects of peristalsis, but this can be justified by noting that (i) stenting generally results in a pronounced reduction of ureteric peristalsis [3-5], and (ii) the accumulation of extracellular collagen in dilated ureters can lead to increased wall stiffness and reduced distensibility [6]. We note, too, that** for the aims of our experiments (to examine the relative times to failure (or not) among the different stent sizes and configurations subject to a reasonable realization of an EUO and a specific colloidal concentration), delineation of the specific mechanical properties of the tubing is not particularly relevant; analysis of responses with ureters (tubing) having different elastic and other properties is beyond the scope of the study.

We note that while **Shilo et al. [1, 2] used a 3.18 mm internal diameter latex tube to simulate the ureter, a 4.76 mm internal diameter was employed in all experiments here (for uniformity) to accommodate the tandem 7F (and endopyelotomy) stents, with total maximum diameter of 4.67 mm. The results of Shilo et al. [2] for the 8F stent in the presence of colloidal solution are relevant in the demonstration that the stent maintains patency: w**hile the relative contributions of stent and ureter lumina to overall flow remain poorly understood, the stent lumen likely controls flow behavior particularly in the vicinity of the EUO where the ureter lumen is (fully or largely) obstructed. Thus, this ultimately affects overall flow through the ureter-stent system. Moreover, the tubing used **in Shilo et al. [2] for the 8F stent was *narrower* than that reported in the current experiments, permitting (relatively) less ureteral flow (i.e., flow in the ureter lumen between the stent and the ureter wall), and yet the 8F stent-ureter system remain free flowing; see [7] for detailed fluid dynamics calculations that quantify this behavior.** To support the behavior proposed here, visual examination of the obstructed stented ureter units in our experiments indicated that (i) colloids accumulated both within the stent (verified by insertion of known length of guidewire that released colloidal aggregates at a specific location, and (ii) in the ureter-stent lumen, at and proximal to the region of compression, as well as further proximal to it. Particle accumulation in a stented ureter around “inactive” stent holes and ureteral “cavities” formed by occlusions was also reported for an *in vitro* experimental study and computational fluid dynamics analysis [8].

*In Vitro EUO Model*

The EUO was simulated by use of a semi-circular compression of length ~3.5 cm located in the region of maximum deformation; a force meter (Lutron, Model FG-5000A) ensured that the extrinsic obstruction against the ureter/stent configuration provided an applied force of 2000 g (≈19.6 N). Clinically, EUO is generally a progressive obstruction; for the purposes of the current study, a static EUO was prescribed.

With regard to the shape of the EUO, the precise structures of EUO and their impact on ureters – and the compressive force that they can exert – remain open questions. As a representative shape, we selected a semi-circular shape to represent an approximately spherical tumor with outer perimeter of 7 cm (≈2 cm diameter). Given the discussion at the end of the previous section, and in the context of the aims of the current study, different choices of EUO shape and size are not expected to have a significant influence. Moreover, in this same context, imposing deformation and compression in regions other that the central region of the ureter are not likely to have any significant effect on the outcome of the experiments (as noted in the main text, Materials and methods section).

Considering the choice of applied force, our previous analyses [1, 2] of ureters containing single polymeric stents (4.8F, 6F, 7F, and 8F stents) demonstrated that **compressive forces up to 5000 g had essentially no effect on fluid flow and stent patency in the 6F, 7F and 8F stents. Patency in the 4.8F stent occurred only above 4000 g compression.** Given that the abdomen contains soft tissue and organs, it seems unlikely that compressive forces acting against a ureter can reach forces of thousands of grams. As such, following a previous study [2], an applied force of 2000 g was chosen. Again, given the discussion at the end of the previous section, and in the context of the aims of the current study, other applied forces (at least up to 4000-5000 g) are not expected to have a significant influence.

*In Vitro Colloidal Solution*

As in Shilo et al. [2], we employed a colloidal solution comprising double distilled water containing chicken albumin from egg white powder (Sigma A5253, molecular weight 44 kDa) at 5 g/L (specific gravity 1.001 at 37 °C). This choice and concentration of **colloidal fluid is not claimed to represent true urine composition, but, rather, was intended to mimic the wide range of suspended organic and inorganic debris – which is largely uncharacterized – in human urine, to assess the impact of colloids on stent failure. Use of chicken albumin as a representative colloid is reported by others [9-11], who also** argued that albumin can represent bacterial and cellular debris found frequently in human urine. **Clearly, many other components (**salts, enzymes and other organic material**) are found in human urine.**

The focus of the current study was to **examine** potential differences in relatively fast stent failure behavior between various stent sizes and configurations, under **the influence of both** ureteral deformation/compression and **colloidal fluid. In this context,** mineralization and deposition mechanisms are generally slower processes than colloid aggregation, extending over weeks to months, and they are thus less likely to account for ubiquitous ureteral stent failure within days due to EUO.

**REFERENCES**

1. Shilo Y, Modai J, Leibovici D, Dror I, Berkowitz B. The impact of ureteral deformation and external ureteral pressure on stent failure in extrinsic ureteral obstruction - An In Vitro experimental study. J Endourol 2020;34(1):68–73.
2. Shilo Y, Modai J, Leibovici D, Dror I, Berkowitz B. [Impact of colloidal fluid on stent failure under extrinsic ureteral obstruction: An In Vitro experimental study](http://dx.doi.org/10.1089/end.2020.0330). J Endourol 2020;34(9):987–992.
3. Kinn AC, Lykkeskov-Andersen H. Impact on ureteral peristalsis in a stented ureter. An experimental study in the pig. Urol Res 2002;30:213–218.
4. Elsamra SE, Leavitt DA, Motato HA, Friedlander JI, Siev M, Keheila M, Hoenig DM, Smith AD, Okeke Z. Stenting for malignant ureteral obstruction: Tandem, metal or metal-mesh stents. Inter J Urol 2015;22:629–636.
5. Liu K-L, Lee B-C, Ye J-D, Chang Y-H, Chang C-C, Huang K-H, Lee Y-J, Chang Y-C. Comparison of single and tandem ureteral stenting for malignant ureteral obstruction: a prospective study of 104 patients. Eur Radiol 2019;29:628–635.
6. Lee BR, Silver RI, Partin AW, Epstein JI, Gearhart JP. A quantitative histologic analysis of collagen subtypes: the primary obstructed and refluxing megaureter of childhood. Urology 1998;51:820–823.
7. Amitay-Rosen T, Nissan A, Shilo Y, Dror I, Berkowitz B: Failure of ureteral stents subject to extrinsic ureteral obstruction and stent occlusions, Int Urol Nephrol, 2021 https://doi.org/10.1007/s11255-021-02810-0
8. Mosayyebi A, Yue QY, Somani BK, Zhang X, Manes C, Carugo D. Particle accumulation in ureteral stents is governed by fluid dynamics: in vitro study using a “stent-on-chip” model. J Endourol 2018;32:639–646.
9. Cox AJ, Hukins DWL, Davies KE, Irlam JC, Sutton TM. An automated technique for in vitro assessment of the susceptibility of urinary catheter materials to encrustation. Eng Med 1987;16(1):37–41.
10. Tunney MM, Bonner MC, Keane PF, Gorman SP. Development of a model for assessment of biomaterial encrustation in the upper urinary tract. Biomaterials 1996;17:1025–1029.
11. Gorman SP, Garvin CP, Quigley F, Jones DS. Design and validation of a dynamic flow model simulating encrustation of biomaterials in the urinary tract. J Pharm Pharmacol 2003;55:461–468.
